# Non-lean versus lean MASLD: associations with advanced cardiovascular-kidney-metabolic syndrome in adults with type 2 diabetes mellitus, a cross-sectional study

**DOI:** 10.3389/fendo.2026.1910398

**Published:** 2026-08-07

**Authors:** Jun-Wei Wang, Le Jiang, Lin-Lin Gao, Jie Ren, Shi-Yu Zhao, Ya-Nan Qiao, Shi-Wei Liu

**Affiliations:** 1 Department of Endocrinology Shanxi Bethune Hospital, Shanxi Academy of Medical Sciences, Third Hospital of Shanxi Medical University, Tongji Shanxi Hospital, The Key Laboratory of Endocrine and Metabolic Diseases of Shanxi Province, Taiyuan, China; 2 Department of Endocrinology, Inner Mongolia Autonomous Region People’s Hospital, Hohhot, China; 3 Department of Orthopedics, Shanxi Bethune Hospital, Shanxi Academy of Medical Sciences, Third Hospital of Shanxi Medical University, Tongji Shanxi Hospital, Taiyuan, China; 4 Department of Pharmacy, Second Hospital of Shanxi Medical University, Taiyuan, China

**Keywords:** CKM syndrome, insulin resistance, lean MASLD, liver fibrosis, non-lean MASLD, visceral fat area

## Abstract

**Introduction:**

This study seeks to explore the impact of lean and non-lean phenotypes of metabolic dysfunction-associated steatotic liver disease (MASLD) on the prevalence of advanced cardiovascular–kidney–metabolic (CKM) syndrome stage in individuals with type 2 diabetes mellitus (T2DM).

**Methods:**

Based on a BMI cutoff of 23 kg/m², 705 hospitalized patients with T2DM and MASLD were split into lean and non-lean categories. Data on clinical characteristics, fat distribution, liver and kidney function, and metabolic markers were collected. The relationship between MASLD phenotypes and concurrent CKM staging severity was evaluated using multivariate logistic regression.

**Results:**

In patients with T2DM and MASLD, 26.7% had lean MASLD. When accounting for age, gender, and diabetes duration, the non-lean MASLD group had higher rates of cardiovascular events (21.3% compared to 10.1%), cerebrovascular events (18.0% compared to 12.2%), and CKM stage 4 (33.5% compared to 18.6%) than the lean group (all P < 0.05). There was no notable difference in the prevalence of CKM stage 3 (61.9% vs. 56.9%, P = 0.148). Binary logistic regression analysis indicated that non-lean MASLD was independently associated with a higher prevalence of advanced stage of CKM, in comparison to lean MASLD (OR = 2.674, P < 0.001). Higher levels of visceral fat area, insulin resistance, and C-peptide were observed in the non-lean group (all P < 0.05), with liver fibrosis indices remaining similar.

**Conclusion:**

In patients with T2DM and MASLD, non-lean MASLD exhibits an independent positive correlation with severe CKM syndrome when compared to lean MASLD.

## Introduction

Metabolic dysfunction-associated steatotic liver disease (MASLD) is a progressive liver condition primarily caused by insulin resistance and metabolic imbalance. Diagnosis requires evidence of liver steatosis and at least one metabolic dysfunction sign (1). MASLD can progress to severe liver issues like cirrhosis and cancer and significantly impacts metabolic health. In those with type 2 diabetes, MASLD affects over 65%, making it the most common chronic comorbidity linked to diabetes (2).

Lean MASLD is defined as MASLD with a BMI under 23.0 kg/m², while non-lean MASLD has a BMI of 23.0 kg/m² or higher, according to Asian-specific criteria. This BMI threshold is recommended in the 2025 APASL MASLD guidelines and supported by research, including a systematic review and a large study of Chinese adults (3–5). Lean MASLD is prevalent in Asian populations, accounting for 23.1% of cases in Chinese and 20.7% in Japanese individuals (6, 7). Despite having a normal BMI, people with lean MASLD have a significantly higher rate of hypertension and diabetes than those without MASLD. A community-based study in Beijing found that people with lean MASLD experienced a 2.3 times higher prevalence of hypertension and a 1.8 times higher prevalence of diabetes compared to those without MASLD (8). Studies show that lean MASLD patients with diabetes exhibit a higher prevalence of advanced liver fibrosis and increased liver-related mortality rates compared to their overweight or obese counterparts (9–11). These findings challenge the belief that “lean MASLD does not require intervention”.

The American Heart Association (AHA) has defined cardiovascular–kidney–metabolic (CKM) syndrome, emphasizing the complex interactions between metabolic risk factors, chronic kidney disease, and cardiovascular disease that lead to multi-organ dysfunction (12). The liver, as a key player in glucose and lipid metabolism, is central to the CKM axis. Metabolic-associated steatotic liver disease (MASLD) can exacerbate CKM syndrome through mechanisms like insulin resistance, chronic inflammation, lipotoxicity, improper RAAS activation, and inter-organ communication (13–15). This has led to the proposal of the renal–hepatic–metabolic (CRHM) syndrome concept (15).

Lean MASLD shows a significant correlation with adverse cardiorenal outcomes. Among hospitalized individuals with MASLD, those classified as lean are at an increased risk for adverse cardiovascular events and renal decompensation during their hospital stay (16). Although some investigations have looked into how lean MASLD relates to non-lean MASLD in comparison with atherosclerotic cardiovascular disease, the results remain controversial. One study reported that lean MASLD patients have a similar risk of cardiovascular events as those who are overweight or obese (17), whereas another study found contrary results, indicating a reduced occurrence of carotid plaques in lean MASLD patients compared to those who are overweight or obese (18). Furthermore, a systematic review and meta-analysis revealed that the risk of chronic kidney disease (CKD) is similar between non-obese and obese MASLD patients, with a pooled odds ratio (OR) of 0.92 (19). In contrast, evidence from the Constances cohort suggested that lean MASLD was linked to a higher CKD prevalence (20). Consequently, the comprehensive effects of lean versus non-lean MASLD on cardiovascular and renal outcomes in CKM syndrome remain insufficiently understood (21). Furthermore, individuals with T2DM exhibit a high prevalence of lean MASLD (22), but the effects of lean MASLD and non-lean MASLD on CKM syndrome in T2DM are not well-documented.

The research conducted a real-world study with 705 hospitalized patients having both MASLD and T2DM. It examines differences between non-lean and lean MASLD patients in CKM stage 3 and 4. The study aims to clarify the independent relationships between MASLD phenotypes and CKM syndrome, supporting early screening and personalized management for T2DM patients with various MASLD types.

## Materials and methods

### Subjects and study design

**Figure 1:**
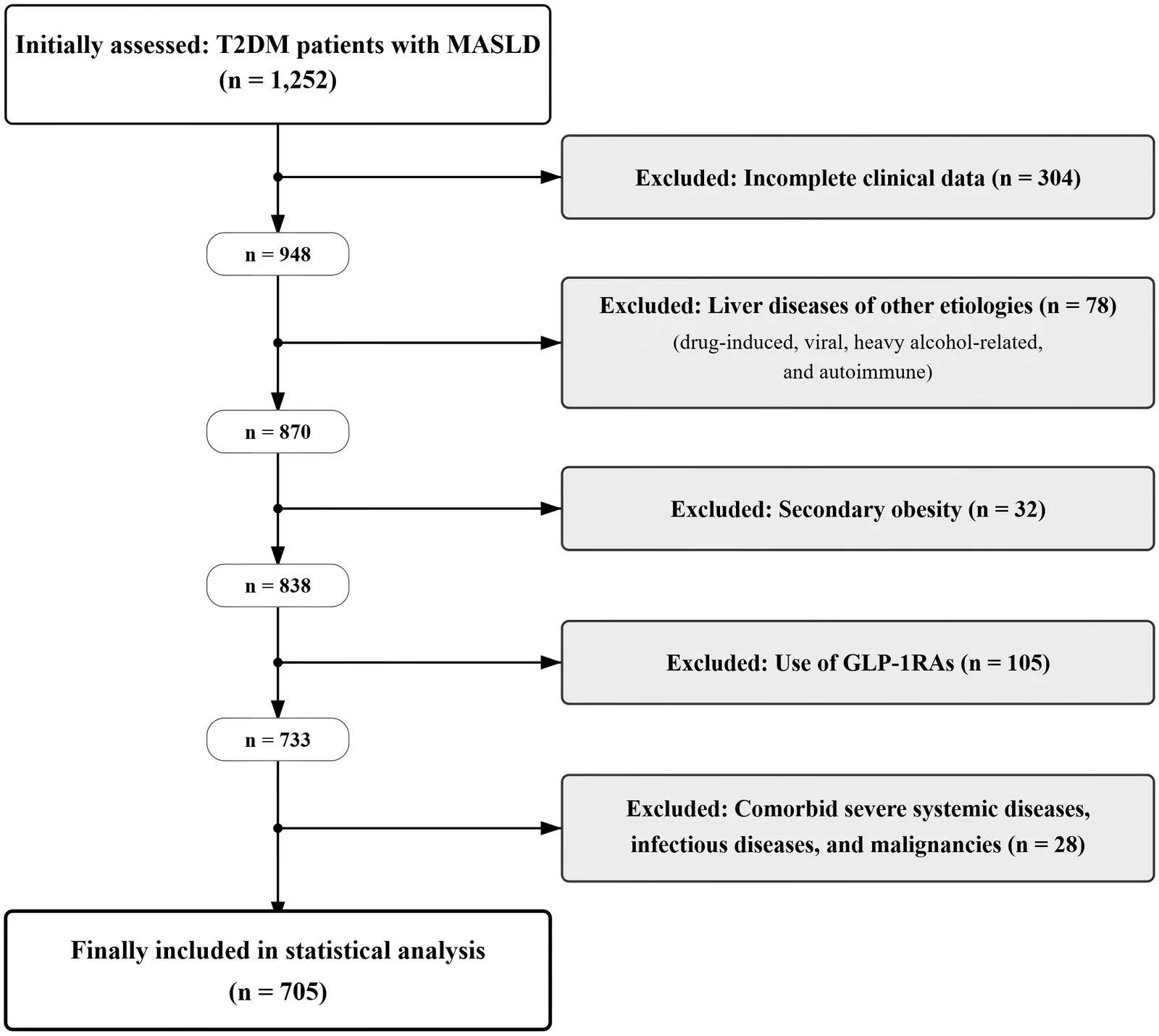
Enrollment of subjects.

This study at Shanxi Bethune Hospital enrolled patients with T2DM and MASLD between January 2020 and June 2021 and was approved by the Ethics Committee of Shanxi Bethune Hospital. Eligibility required participants to be 18 or older, have T2DM, ultrasound-confirmed hepatic steatosis, and complete clinical data. Exclusions were other liver diseases, secondary obesity, glucagon-like peptide-1 receptor agonists (GLP-1RAs) use, severe systemic or infectious diseases, malignancies, and incomplete data. The analysis included 705 patients, categorized into lean MASLD (BMI < 23 kg/m²) and non-lean MASLD (BMI ≥ 23 kg/m²) based on Asian MASLD guidelines (4). See **Figure 1** for the flow diagram.

### Clinical data

Upon admission, the following clinical data were collected: history of hypertension, dyslipidemia, duration of diabetes (DD), alcohol intake, smoking behaviors, and the use of lipid-lowering drugs (LLDs), oral antidiabetic drugs (OADs), and insulin or insulin analogs (IIAs) (23). Smoking and alcohol consumption were categorized as follows: Current smokers are those who smoke at least one cigarette daily for a total of six months or more, including the past year. Former smokers met this criterion but have not smoked for at least six months before enrollment. Current drinkers consumed alcohol at least weekly for six consecutive months within the last 6–12 months. Former drinkers met this criterion but have abstained from alcohol for at least six months before enrollment (24).

### Physical examination

Measurements were taken for the patients’ height, weight, systolic blood pressure (SBP), and diastolic blood pressure (DBP), and BMI was determined by dividing weight in kilograms by the square of height in meters. The Omron DUALSCAN HDS-2000 bioelectrical impedance analyzer was employed to determine the subcutaneous fat area (SFA) and visceral fat area (VFA). All assessments were conducted by certified physicians, and patients were mandated to fast for a minimum of 12 hours prior to the testing procedures.

### Laboratory tests

On the morning of the second day after admission, venous blood and urine samples were collected following a fasting period of more than 12 hours. The subsequent analyses included the evaluation of the following parameters: glycosylated hemoglobin (HbA1c), alanine aminotransferase (ALT), aspartate aminotransferase (AST), total cholesterol (TC), triglycerides (TG), high-density lipoprotein cholesterol (HDL-C), low-density lipoprotein cholesterol (LDL-C), platelet count (PLT), albumin (ALB), serum creatinine (Cr), serum uric acid (SUA), and the ratio of urinary albumin to creatinine (UACR). Additionally, all patients underwent a 75 g oral glucose tolerance test, with blood samples taken while fasting and at 30, 60, and 120 minutes after glucose intake to evaluate blood glucose and C-peptide concentrations. The glucose and C-peptide area under the curve (AUC) was determined utilizing the trapezoidal method, employing the following formulas: Glucose AUC = [(glucose_0min_ + glucose_30min_)/2 × 30] + [(glucose_30min_ + glucose_60min_)/2 × 30] + [(glucose_60min_ + glucose_120min_)/2 × 60]; C-peptide AUC = [(C-peptide_0min_ + C-peptide_30min_)/2 × 30] + [(C-peptide_30min_ + C-peptide_60min_)/2 × 30] + [(C-peptide_60min_ + C-peptide_120min_)/2 × 60]. High-sensitivity cardiac troponin I (hs-cTnI) levels were measured with a two-site enzyme immunoassay, while B-type natriuretic peptide (BNP) concentrations were assessed via electrochemiluminescence. To compute the insulin resistance index (HOMA2-IR), the HOMA2-IR calculator found at http://www.dtu.ox.ac.uk was utilized. Estimated glomerular filtration rate (eGFR) was calculated using the CKD-EPI equation, including the updated 2021 CKD-EPI formulation without race coefficients (25). To determine the severity of liver fibrosis, the FIB-4 index was calculated as (age × AST)/(PLT × √ALT). FIB-4 index was calculated for all subjects to screen for advanced hepatic fibrosis. Consistent with AASLD guidance (26), FIB-4 > 2.67 was adopted as the uniform diagnostic cutoff for advanced fibrosis for patients of all age groups.

### Ultrasonography

Two skilled sonographers used a 5–13 MHz transducer with the Philips EPIQ 7 system for carotid Doppler ultrasonography to evaluate blood vessel morphology and flow. They measured intima-media thickness and checked for atherosclerotic plaques at various sites of the common, bifurcation, external, and internal carotid arteries in both transverse and longitudinal views. Abdominal ultrasonography, with a 3.5 MHz probe through the right intercostal space, was conducted to diagnose hepatic steatosis by detecting increased liver echogenicity, intrahepatic vascular blurring, or deep ultrasound signal attenuation (23).

### Diagnostic criteria

CKM stage 3 involves either subclinical cardiovascular damage or very high-risk CKD without overt cardiovascular disease (CVD). Diagnosis requires one of the following: Subclinical cardiovascular damage: - Carotid intima-media thickness of 0.9 mm or more, or carotid plaques. - hs-cTnI above the 99th percentile without acute myocardial infarction. - Elevated BNP without heart failure. Very high-risk CKD: - eGFR below 30 mL/min/1.73 m². - eGFR between 30–59 mL/min/1.73 m² with UACR over 300 mg/g (12).

CKM stage 4 is marked by confirmed clinical cardiovascular or cerebrovascular disease at any CKD stage. CVD includes coronary artery disease, heart failure, atrial fibrillation without valve issues, significant peripheral artery narrowing, or previous peripheral vascular surgery. CBVD is defined as stroke verified on cranial imaging or transient ischemic attack diagnosed by neurologists with corresponding imaging evidence (12).

In this study on T2DM patients, MASLD was identified through ultrasound-detected hepatic steatosis and confirmed T2DM (27). Hypertension was defined by an SBP of at least 140 mmHg, a DBP of at least 90 mmHg, or ongoing treatment for hypertension. Dyslipidemia was diagnosed based on TG levels of 1.7 mmol/L or higher, LDL-C levels of 3.37 mmol/L or higher, TC levels of 5.18 mmol/L or higher, HDL-C levels below 1.04 mmol/L, or the use of lipid-lowering medications (23).

### Statistical analysis

Data analysis was conducted using the full-functional 14-day trial version of IBM SPSS Statistics 32.0. The Kolmogorov–Smirnov test assessed normality for continuous variables. Normally distributed data are shown as mean ± standard deviation and analyzed with t-tests, while non-normally distributed data are presented as median (interquartile range) and analyzed with the Mann–Whitney U test. Categorical data are expressed as counts (percentages) and compared using the chi-square test. Binary logistic regression was used for categorical outcomes and univariate linear regression for continuous outcomes, adjusting for sex, age, and DD. Group-specific 95% Wilson score confidence intervals for each outcome were calculated using one-sample proportion analysis. Binary logistic regression models, with lean MASLD as the reference, assessed associations with concurrent CKM syndrome status, using three models with stepwise adjustments: Model 1 included age, gender, DD, smoking, alcohol, dyslipidemia, and hypertension; Model 2 added medications, blood pressure, and fat areas; Model 3 included biochemical markers. Statistical significance was set at P < 0.05, with P values from 0.05 to 0.10 indicating a trend.

## Results

### The study subjects’ characteristics

The study examined 705 hospitalized patients with T2DM and MASLD, divided into lean and non-lean groups (**Table 1**). After adjusting for age and sex, the non-lean group showed significantly higher SBP, DBP, C-peptide_0min_, C-peptide_120min_, TG, ALT, Cr, and SFA, and lower HDL-C compared to the lean group (P<0.05). They also had higher rates of hypertension and dyslipidemia, and increased TC, LDL-C, and UACR, with near-significant differences (0.05<P<0.10). No significant differences were found between the groups in DD, smoking, alcohol use, LLDs, OADs, IIAs, or in HbA1c, glucose_0min_, glucose_120min_, AST, PLT, ALB, hs-cTnI, BNP, eGFR, and SUA levels.

**Table 1:** Baseline characteristics of the study population (lean vs non-lean MASLD).

Variables	Lean MASLD (n=188)	Non-lean MASLD (n=517)	Unadjusted P values	Adjusted P value
Age, years	55.8 ± 12.7	53.3 ± 12.4	0.018	0.038
Male, n (%)	74 (39.4)	245 (47.4)	0.06	0.131
*DD, years	7.0 (2.5-13.0)	7.1 (2.0-12.0)	0.375	0.498
BMI, kg/m²	21.2 ± 1.4	26.8 ± 3.0	<0.001	<0.001
Hypertension, n (%)	95 (50.5)	290 (56.1)	0.19	0.078
SBP, mmHg	131 ± 19	137 ± 18	0.001	0.023
DBP, mmHg	77 ± 11	81 ± 12	<0.001	0.005
Dyslipidemia, n (%)	81 (43.0)	268 (51.8)	0.04	0.052
Smoking history, n (%)	56 (29.8)	167 (32.3)	0.583	0.292
Alcohol consumption history, n (%)	36 (19.1)	103 (19.9)	0.915	0.426
LLDs, n (%)	79 (42.0)	228 (44.1)	0.62	0.841
OADs, n (%)	141 (75.0)	362 (78.4)	0.468	0.481
IIAs, n (%)	57 (30.3)	166 (32.1)	0.714	0.321
HbA1C, %	9.1 ± 2.5	8.9 ± 2.1	0.407	0.201
* glucose_0min_ (mmol/l)	7.76 (6.26-9.72)	7.93 (6.64-9.80)	0.047	0.157
*glucose_120min_ (mmol/l)	17.60 (14.00-20.44)	18.55 (15.35-21.67)	0.407	0.351
* C-peptide_0min_ (ng/mL)	2.35 (1.22-7.66)	2.65 (1.70-5.03)	<0.001	0.019
*C-peptide_120min_ (ng/mL)	3.61 (2.62-5.98)	5.18 (3.48-7.48)	0.002	0.012
*TG (mmol/l)	1.41 (0.98-2.19)	1.88 (1.31-2.97)	<0.001	0.001
TC (mmol/l)	4.53 ± 1.16	4.73 ± 1.37	0.061	0.087
HDL-C (mmol/l)	1.20 ± 0.31	1.09 ± 0.29	<0.001	<0.001
LDL-C (mmol/l)	2.70 ± 0.76	2.83 ± 0.81	0.069	0.057
*ALT (U/l)	21.3 (13.0-28.6)	23.9 (16.2-39.2)	<0.001	0.002
*AST (U/l)	18.5 (16.0-25.5)	20.0 (16.3-28.2)	0.298	0.53
*PLT (10^9/L)	202 (171–240)	207 (171–249)	0.844	0.958
*ALB (g/L)	41.1 (38.7-43.4)	41.0 (39.2-43.3)	0.968	0.498
*hs-cTnI (pg/ml)	6.9 (5.6-9.5)	8.2 (6.4-10.9)	0.710	0.833
*BNP (pg/ml)	19.6 (12.4-27.1)	18.9 (11.8-26.5)	0.474	0.529
*SFA (cm^2^)	121.4 (96.6-153.2)	204.8 (171.2-239.1)	<0.001	<0.001
*Cr (μmol/l)	67.4 (60.5-74.7)	69.4 (63.4-77.4)	0.003	0.037
eGFR, mL/min/1.73m²	94.22 (83.16-108.97)	98.10 (83.40-106.90)	0.232	0.127
*SUA (μmol/l)	265 (216–338)	342 (275–400)	<0.001	0.193
*UACR, mg/g	11.5 (7.4-23.2)	13.4 (8.5-38.7)	0.025	0.057

Data are presented as mean ± SD, median with interquartile range, or percentages. Unadjusted P values were not adjusted for age and sex for the trend. Adjusted P values were adjusted for sex and age for the trend. Gender-based comparisons were adjusted only for age, and age-based comparisons were adjusted only for sex. The adjusted P values should be considered the primary comparisons between groups. *The Kruskal-Wallis test was applied.

### Comparison of the prevalence of CKM stage 3 and its components between the two groups

**Figure 2:**
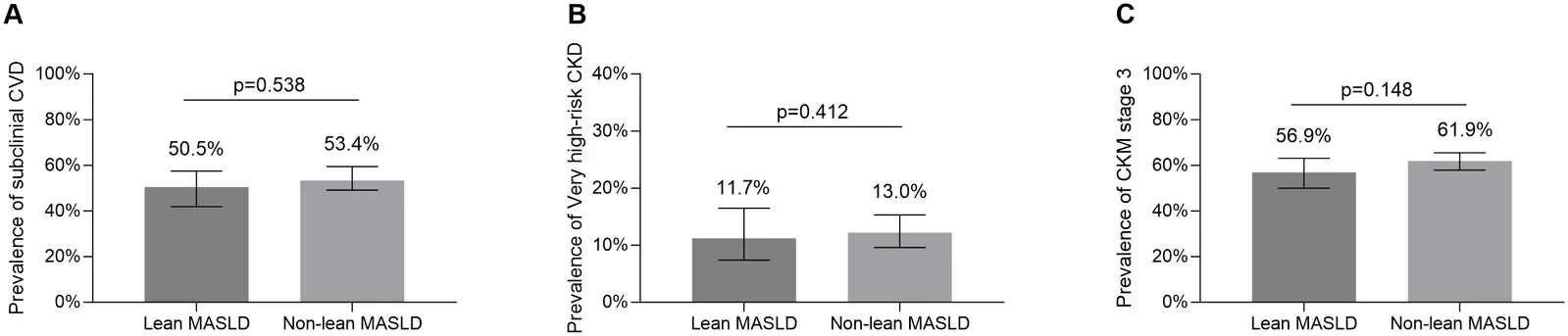
Comparison of the prevalence of CKM stage 4 and its components between the two groups. **(A)** Comparison of clinical CVD prevalence between groups, adjusted for age, sex, and DD. **(B)** Comparison of CBVD prevalence between groups, adjusted for age, sex, and DD. **(C)** Comparison of CKM syndrome stage 4 prevalence between groups, adjusted for age, sex, and DD. Bars show the prevalence proportion of each indicator; error bars represent 95% Wilson score confidence intervals.

**Figure 2** compares the prevalence of subclinical cardiovascular damage, very high-risk CKD, and CKM syndrome stage 3 between lean and non-lean MASLD groups after adjusting for sex, age, and DD. Subclinical cardiovascular damage was found in 50.5% of the lean group and 53.4% of the non-lean group (P = 0.538), very high-risk CKD in 11.7% and 13.0% respectively (P = 0.412), and CKM syndrome stage 3 in 56.9% and 61.9% respectively (P = 0.148). None of these differences were statistically significant.

### Comparison of the prevalence of CKM stage 4 and its components between the two groups

**Figure 3:**
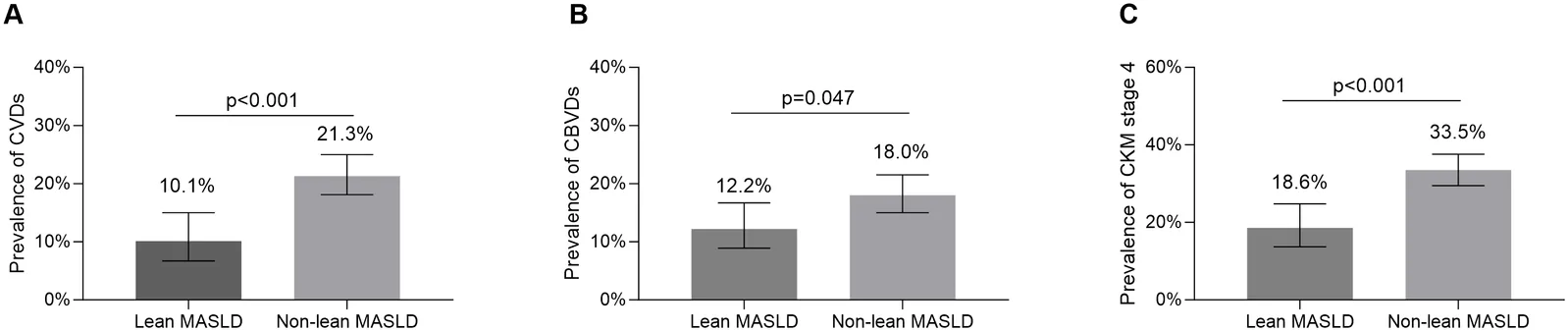
Comparison of the prevalence of CKM stage 3 and its components between the two groups. **(A)** Compare subclinical cardiovascular damage prevalence between the groups, adjusted for age, sex, and DD. **(B)** Compare very high-risk CKD prevalence between the groups, adjusted for age, sex, and DD. **(C)** Compare CKM syndrome stage 3 prevalence between the groups, adjusted for age, sex, and DD. Bars show the prevalence proportion of each indicator; error bars represent 95% Wilson score confidence intervals.

**Figure 3** compares the prevalence of CVDs, CBVDs, and CKM stage 4 between two groups after controlling for sex, age, and DD. Clinical cardiovascular events were significantly lower in the lean MASLD group (10.1%) than in the non-lean group (21.3%, P<0.001). Cerebrovascular events were also less common in the lean group (12.2%) compared to the non-lean group (18.0%, P = 0.047). CKM stage 4 was found in 18.6% of the lean group, significantly lower than the 33.5% in the non-lean group (P<0.001).

### Comparison of fat distribution, liver fibrosis, insulin resistance and glucose metabolism between the two groups

**Figure 4:**
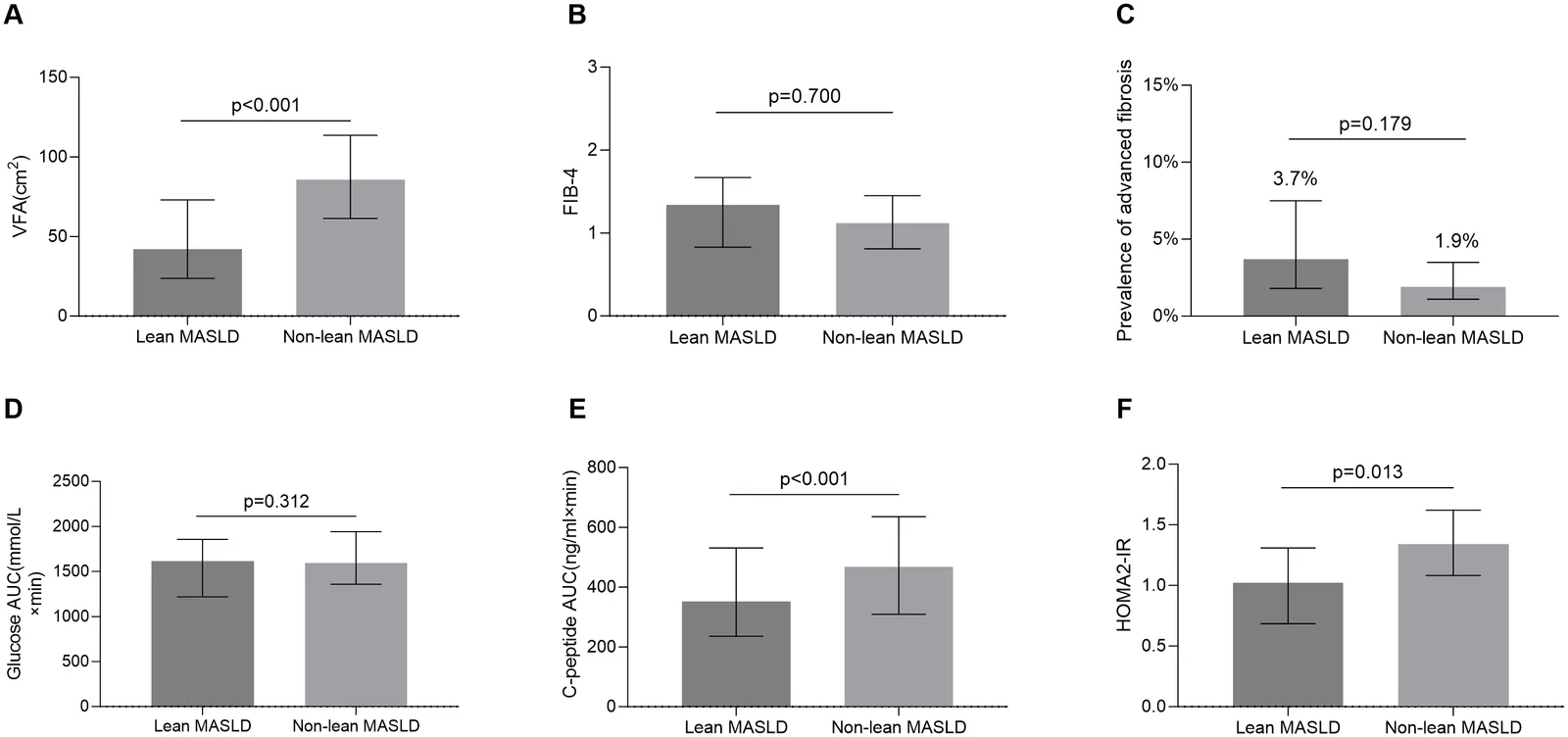
Comparison of fat distribution, liver fibrosis, glucose metabolism, and insulin resistance indicators between lean and non-lean MASLD patients. **(A)** VFA; **(B)** FIB-4; **(C)** Liver fibrosis prevalence. Bars show the prevalence proportion of liver fibrosis and error bars represent 95% Wilson score confidence intervals; **(D)** Glucose AUC; **(E)** C-peptide AUC; **(F)** HOMA2-IR. All comparisons adjusted for age, sex, and DD.

We performed a comparative analysis of fat distribution, severity of liver fibrosis, glucose metabolism, and markers of insulin resistance between two groups, with adjustments made for age, sex, and DD. The VFA was significantly greater in patients with non-lean MASLD compared to their lean counterparts (lean group: median 42 cm², interquartile range [IQR] 24–73 cm²; non-lean group: median 86 cm², IQR 61–114 cm²; P < 0.001). No significant differences were observed between the groups regarding FIB-4 scores (lean group: median 1.34, IQR 0.83–1.67; non-lean group: median 1.12, IQR 0.81–1.45), the prevalence of advanced hepatic fibrosis, or glucose area under the curve (AUC) (lean group: median 1617 mmol-min/L, IQR 1219–1857 mmol-min/L; non-lean group: median 1595 mmol-min/L, IQR 1358–1941 mmol-min/L). However, the non-lean MASLD group exhibited significantly higher C-peptide AUC (lean group: median 353 ng-min/L, IQR 236–531 ng-min/L; non-lean group: median 468 ng-min/L, IQR 309–636 ng-min/L; P < 0.001) and homeostasis model assessment of insulin resistance (HOMA2-IR) scores (lean group: median 1.023, IQR 0.684–1.309; non-lean group: median 1.341, IQR 1.082–1.621; P = 0.013). These results are presented in **Figure 4**.

### Correlation analysis of CKM staging status between lean and non-lean MASLD patients

Taking the lean MASLD population as the reference group, we constructed three multivariable logistic regression models with stepwise adjustment for confounders to evaluate the independent correlations of non-lean MASLD with concurrent CKM stage ≥3 and concurrent CKM stage 4 (**Table 2**). In Model 1 adjusted for demographic and lifestyle indicators, non-lean MASLD showed a robust correlation with the presence of CKM stage 3 or above (OR = 1.801, P = 0.005) and the presence of CKM stage 4 (OR = 2.499, P<0.001). After further adjusting for medication history, blood pressure and fat area in Model 2, non-lean MASLD still correlated with the presence of CKM stage 4 (OR = 2.597, P = 0.003), and the correlation with CKM stage ≥3 status retained statistical significance (OR = 1.749, P = 0.044). Model 3 underwent full adjustment for all confounding covariates including lipid profiles, hepatic fibrosis, glycemic status and insulin resistance; non-lean MASLD exhibited an independent correlation with CKM stage 4 status, with the OR value approaching twice that of the reference group (OR = 2.674, P<0.001). By contrast, the statistical correlation between non-lean MASLD and CKM stage 3 or above status disappeared (OR = 1.624, P = 0.319).

**Table 2:** Comparison of the ORs for CKM syndrome between lean and non-lean MASLD patients with T2DM.

	ORs (95% CI)		P value
Variables	Lean MASLD	Non-lean MASLD	
CKM stage≥3			
Model 1	1	1.801 (1.196-2.712)	0.005
Model 2	1	1.749 (1.016-3.013)	0.044
Model 3	1	1.624 (0.626-4.217)	0.319
CKM stage 4			
Model 1	1	2.499 (1.618-3.858)	<0.001
Model 2	1	2.597 (1.378-4.894)	0.003
Model 3	1	2.674 (1.532-4.668)	<0.001

Model 1: Adjusted for age, sex, DD, smoking status, alcohol intake, dyslipidemia and hypertension.

Model 2: Further adjustment for use of LLDs, IIAs, OADs, SBP, DBP, and VFA.

Model 3: Further adjustment for TG, HDL-C, LDL-C, SUA, FIB-4 and HOMA2-IR.

### Sensitivity analysis

A sensitivity analysis using three models examined the link between non-lean MASLD (BMI ≥25 kg/m²) and CKM syndrome in T2DM patients, with lean defined as BMI <25 kg/m² (**Supplementary Table 1**). The association between non-lean status and CKM stage ≥3 vanished after adjusting for covariates. However, the risk of CKM stage 4 with non-lean MASLD remained significant across all models, confirming its stability despite metabolic confounders. Attenuation analysis showed VFA and HOMA2-IR mediated 13.72% and 24.80% of the non-lean MASLD and stage 4 CKM link, respectively, highlighting insulin resistance as the main pathway (**Supplementary Table 2**). Joint adjustment of these factors resulted in a 15.99% attenuation, indicating overlapping pathways where visceral fat worsens renal damage via insulin resistance. The combined factors accounted for less than 16% of the overall risk difference. Sex-stratified analyses explored the relationship between non-lean MASLD and CKM syndrome (**Supplementary Table 3**). For CKM stage ≥3, initial significant associations in both sexes lost significance after adjusting for covariates. However, for severe CKM stage 4, the heightened risk linked to non-lean MASLD remained significant across all models for both genders.

## Discussion

The study revealed a prevalence of 26.7% for lean MASLD among T2DM patients, similar to the 23.1% reported in the Chinese population (6). Given that the prevalence of MASLD is approximately 50% among individuals with T2DM, the observed prevalence of lean MASLD in this study may be regarded as comparatively high (22). Although this study identified no significant sex differences in the prevalence of lean MASLD, a retrospective study conducted in China revealed that women with lean MASLD exhibited a higher incidence of metabolic syndrome and experienced more adverse effects compared to their male counterparts (28). Therefore, we conducted sex-stratified analyses and found no significant sex differences in the primary outcomes of the present study. Furthermore, lean MASLD individuals tend to be of relatively advanced age. Large-scale population-based physical examination studies from China indicated that the peak age for lean MASLD was 55–65 years, suggesting that older adults are more susceptible to lean MASLD (29).

CKM syndrome combines metabolic disorders, kidney damage, and cardiovascular disease into a single continuum, enabling comprehensive management from risk factors to terminal events (12). Nevertheless, the differences in the prevalence of CKM syndrome between lean and non-lean MASLD populations remain ambiguous, and there is a paucity of research specifically focusing on individuals with T2DM. A meta-analysis of 21 studies indicated that lean MASLD is associated with a 50% increased prevalence of cardiovascular mortality compared to non-lean MASLD (30). In contrast, a pooled analysis of three prospective cohort studies found that lean MASLD is associated with a lower overall risk of incident CVD compared to non-lean MASLD (31). In this context, the present study systematically examines the distribution of mild to severe CKM staging status in lean versus non-lean MASLD patients with T2DM.

The study found no significant differences between two groups in terms of subclinical cardiovascular injury, very high-risk CKD, or CKM stage 3 prevalence. However, compared to lean MASLD group, the non-lean MASLD group displayed higher concurrent proportions of clinical CVDs, CBVDs, and composite CKM presentations. Even after adjusting for confounders, non-lean MASLD maintained an independent cross-sectional correlation with the presence of CKM stage 4 in individuals with T2DM. These findings provide new insights into the debated link between lean MASLD and clinically evident cardiovascular manifestations, as previous research mainly focused on cardiovascular events without distinguishing between subclinical and clinical CKM stages. This study suggests that MASLD may trigger CKM syndrome by causing subclinical cardiorenal damage, even in people with normal BMI, through insulin resistance and chronic inflammation. As a result, CKM stage 3 is equally common in both lean and non-lean individuals, masking obesity’s effects. Previous research has shown that patients with lean MASLD have elevated coronary artery calcium scores and an increased carotid plaque burden in comparison to individuals without MASLD (32, 33). In this study, over half of lean MASLD patients showed subclinical cardiorenal injury, supporting previous findings. In non-lean MASLD patients, visceral fat accumulation linked to obesity and elevated insulin resistance correlate with the presence of clinical organ damage rather than only subclinical lesions, alongside concurrent CKM stage 4 status. Results from prospective cohort studies corroborate our findings, indicating that within the general population, lean MASLD is associated with a reduced overall risk of developing cardiovascular disease compared to non-lean MASLD (HR 0.89, 95% CI 0.83–0.95) (31). This study, which concentrates on a cohort with T2DM exhibiting greater metabolic dysfunction, underscores the substantial influence of obesity on end-stage CKM, providing novel insights for risk stratification across diverse populations.

In our cohort of 17 patients with advanced hepatic fibrosis, the comparison of fibrosis-related indices between lean and non-lean MASLD groups revealed only a borderline statistical trend, without significant intergroup differences. This finding may partially elucidate the relationship between MASLD phenotypes and CKM stage. Prior research show that lean MASLD patients are more likely to have progressive liver fibrosis, which may serve as an independent contributor to adverse cardiovascular outcomes (17, 20). Nonetheless, our study identified that T2DM patients with non-lean MASLD exhibited significantly higher odds of advanced CKM stage compared to those with lean MASLD. In light of the absence of significant differences in FIB-4 values between groups, the increased prevalence of advanced CKM observed in the non-lean MASLD group may be largely independent of the severity of liver fibrosis. Furthermore, despite constituting a minor proportion of total body fat, visceral adipose tissue is hormonally active and secretes pro-inflammatory cytokines such as interleukin-6 (IL-6) and tumor necrosis factor-alpha (TNF-α). These secretions contribute to chronic inflammation, endothelial dysfunction, and insulin resistance, thereby impacting cardiovascular and renal health via hepatic pathways (9, 34). Individuals with lean MASLD, even with a normal BMI, often have hidden visceral fat, leading to higher rates of subclinical cardio-renal injury (35). This study confirms that non-lean MASLD patients with more visceral fat tend to have higher odds of CKM stage 4 when fibrosis severity is similar.

Insulin resistance is a key factor in the development of CKM syndrome and explains the differences in CKM risk between the groups studied. The study shows that the non-lean MASLD group has higher C-peptide AUC and HOMA2-IR index levels than the lean MASLD group, indicating greater insulin resistance and hyperinsulinemia. In T2DM patients, insulin resistance in liver and fat tissues is common, with visceral fat accumulation worsening systemic insulin resistance, leading to a harmful cycle of “visceral fat accumulation - insulin resistance - hepatic steatosis - increased insulin resistance.” Chronic high blood sugar and insulin levels due to insulin resistance can harm blood vessels, activate inflammation, and worsen atherosclerosis. These conditions also damage kidney cells, advancing CKM syndrome from early damage to noticeable symptoms (36). **Supplementary Table 2** shows that insulin resistance has a greater mediating effect than visceral fat on advanced stage 4 CKM syndrome. The shared pathways of these two metabolic factors limit their combined explanatory power, suggesting other unmeasured mechanisms may be involved. In the CKM≥3 stage group, VFA is closely linked to renal impairment and subclinical atherosclerosis, influencing Model 2. Without VFA adjustment, Model 2 considers the overlap between visceral fat, renal injury, and vascular lesions through metabolic and blood pressure covariates, slightly weakening the non-lean MASLD–CKM link. Including VFA separately captures its variance, increasing the OR and causing negative attenuation.

The study found that individuals with lean MASLD showed metabolic benefits, including lower rates of hypertension, reduced blood pressure, and higher HDL-C levels, which lower the risk of CKM end-stage events. HDL-C supports cardiovascular and kidney health through reverse cholesterol transport, improved endothelial function, and reduced inflammation. In contrast, hypertension damages heart and kidney organs, with RAAS overactivation being a common pathological link (37, 38). Several studies have shown that lean MASLD patients typically have better blood pressure and lipid profiles than their obese counterparts, which helps reduce the risk of CKM associated with hepatic steatosis (18, 39). These factors may partially explain why a larger proportion of patients with non-lean MASLD presented with stage 4 CKM syndrome are relative to those with lean MASLD in the present study.

The current study offers corroborative evidence for the management of CKM syndrome in patients with T2DM who also have MASLD. Our findings indicate that over 50% of T2DM patients with MASLD, irrespective of being lean or non-lean, exhibited stage 3 CKM syndrome. Consequently, it is recommended that all T2DM patients diagnosed with MASLD be prioritized for routine screening for CKM syndrome. This should include regular carotid ultrasonography and the assessment of cardiac and renal injury biomarkers, along with the early administration of cardiorenal protective agents (40). Notably, patients with non-lean MASLD demonstrated a 1.67-fold higher odds of concurrent stage 4 CKM syndrome relative to patients with lean MASLD, suggesting a significant association between non-lean MASLD and advanced CKM lesions. Therefore, clinicians should be particularly vigilant regarding the risk of severe CKM syndrome in non-lean MASLD patients. Strategies such as weight management and reduction of visceral fat, alongside improvements in insulin resistance, normalization of dyslipidemia, and control of hypertension, can effectively mitigate the risk of advanced CKM syndrome.

This study is subject to certain limitations. Firstly, it was designed as a cross-sectional study, which inherently limits its capacity to discern only associations between various phenotypes of MASLD and CKM syndrome, without establishing causality or tracking the progression of CKM syndrome over time. Secondly, MASLD was diagnosed using standard abdominal ultrasound, a technique that is less sensitive compared to advanced methods such as MRI-proton density fat fraction or transient elastography. The use of ultrasound may fail to detect mild steatosis, particularly in lean individuals, which could lead to the misclassification of some patients as non-MASLD. This non-differential misclassification may impact the categorization of lean versus non-lean MASLD and likely results in conservative estimations of the association between MASLD phenotypes and CKM stage. Thirdly, the estimation of visceral fat area in this study was conducted using BIA rather than more precise imaging techniques such as CT or MRI. BIA measurements are susceptible to variability due to factors such as hydration status, recent food or fluid intake, and the algorithms employed by the device, which may introduce measurement errors. Fourthly, the study cohort comprised hospitalized patients with T2DM who were recruited during the COVID-19 pandemic. This recruitment period may have introduced a bias towards individuals with multiple chronic conditions. During this time, stringent anti-epidemic measures were implemented across China, and patients who tested positive for COVID-19 were excluded from the study. Consequently, caution should be exercised when generalizing these findings to community-based populations or non-pandemic contexts. Finally, the study excluded 105 patients who were already receiving GLP-1RAs at the time of enrollment. Given that GLP-1RAs are predominantly prescribed for individuals with severe obesity and metabolic complications, their exclusion may impact the external validity of the cohort, particularly concerning high-risk T2DM patients undergoing intensive treatment.

## Conclusion

This study shows that in T2DM patients with MASLD, the non-lean phenotype of MASLD is independently correlated with increased concurrent detection rates of CKM syndrome. These associations may be explained by visceral fat accumulation and insulin resistance. It highlights the link between MASLD phenotypes and CKM syndrome in T2DM patients, indicating even lean MASLD patients also present elevated concurrent prevalence of subclinical cardiovascular and renal damage. These findings support early risk assessment and personalized management for CKM syndrome in this group.

## Data Availability

The raw data supporting the conclusions of this article will be made available by the authors, without undue reservation.
