# Supplementary material for: Non-lean versus lean MASLD: associations with advanced cardiovascular-kidney-metabolic syndrome in adults with type 2 diabetes mellitus, a cross-sectional study
Source: Front Endocrinol (Lausanne). 2026 Aug 7;17:1910398. doi: 10.3389/fendo.2026.1910398 (PMC13493292; doi:10.3389/fendo.2026.1910398)
Supplement: Supplementary file 1 [file Table1.docx]

**Table S1 Comparison of the ORs for CKM Syndrome Between Lean (BMI <25kg/m^2^) and Non-lean (BMI ≥25kg/m^2^) MASLD Patients with T2DM**

|  | ORs (95% CI) | | | | P value |
| --- | --- | --- | --- | --- | --- |
|  | Lean MASLD | Non-lean MASLD | | |  |
| CKM stage≥3 |  |  |  |  |  |
| Model 1 | 1 | 1.490(1.024-2.170) | | | 0.037 |
| Model 2 | 1 | 1.291(0.772-2.160) | | | 0.330 |
| Model 3 | 1 | 1.325(0.539-3.261) | | | 0.540 |
| CKM stage 4 |  |  |  |  |  |
| Model 1 | 1 | 2.034(1.427-2.900) | | | <0.001 |
| Model 2 | 1 | 1.652(1.009-2.706) | | | 0.046 |
| Model 3 | 1 | 1.759 (1.056-2.930) | | | 0.030 |

Model 1: Adjusted for age, sex, DD, smoking status, alcohol intake, dyslipidemia and hypertension.

Model 2: Further adjustment for use of LLDs, IIAs, OADs, SBP, DBP, and VFA.

Model 3: Further adjustment for TG, HDL-C, LDL-C, SUA, FIB-4 and HOMA2-IR.
