# Supplementary material for: Non-lean versus lean MASLD: associations with advanced cardiovascular-kidney-metabolic syndrome in adults with type 2 diabetes mellitus, a cross-sectional study
Source: Front Endocrinol (Lausanne). 2026 Aug 7;17:1910398. doi: 10.3389/fendo.2026.1910398 (PMC13493292; doi:10.3389/fendo.2026.1910398)
Supplement: Supplementary file 2 [file Table2.docx]

**Table S2 Sensitivity Analysis: ORs for CKM Syndrome Comparing Lean and Non-lean Patients with MASLD and T2DM via Multivariable Adjustment**

|  | ORs (95% CI) | | | | P value | Attenuation proportion (%) |
| --- | --- | --- | --- | --- | --- | --- |
|  | Lean MASLD | Non-lean MASLD | | |  |  |
| CKM stage≥3 |  |  |  |  |  |  |
| Model 1 | 1 | 1.801(1.196-2.712) | | | 0.005 | — |
| Model 2 | 1 | 1.697(1.116-2.580) | | | 0.013 | Reference |
| Model 3 | 1 | 1.749(1.016-3.013) | | | 0.044 | −7.46 |
| Model 4 | 1 | 1.569(0.872-2.823) | | | 0.133 | 18.36 |
| Model 5 | 1 | 1.634(0.941-2.839) | | | 0.081 | 9.04 |
| Model 6 | 1 | 1.624(0.626-4.217) | | | 0.319 | — |
| Model 7 | 1 | 1.987(0.682-5.791) | | | 0.208 | — |
| CKM stage 4 |  |  |  | — |  |  |
| Model 1 | 1 | 2.499(1.618-3.858) | | | <0.001 | — |
| Model 2 | 1 | 2.851(1.819-4.467) | | | ＜0.001 | Reference |
| Model 3 | 1 | 2.597 (1.378-4.894) | | | 0.003 | 13.72 |
| Model 4 | 1 | 2.392(1.332-4.297) | | | 0.004 | 24.8 |
| Model 5 | 1 | 2.555(1.353-4.823) | | | 0.004 | 15.99 |
| Model 6 | 1 | 2.674 (1.532-4.668) | | | <0.001 | — |
| Model 7 | 1 | 2.573(1.276-5.188) | | | 0.008 | — |

Model 1: Adjusted for age, sex, DD, smoking status, alcohol intake, dyslipidemia, and hypertension.

Model 2: Model 1 + LLDs, IIAs, OADs, SBP, DBP.

Model 3: Model 2 + VFA.

Model 4: Model 2 + HOMA2-IR.

Model 5: Model 2 + VFA + HOMA2-IR.

Model 6: Model 5 + TG, HDL-C, LDL-C, SUA, FIB-4.

Model 7: Model 3 + TG, HDL-C, LDL-C, SUA, FIB-4. (without HOMA2-IR)

Attenuation proportion was calculated to quantify the share of association mediated by VFA and HOMA2-IR, using the formula:Attenuation proportion = (OR of reference Model 2 − OR of mediator-adjusted model) / (OR of reference Model 2 − 1).Model 2 was set as the reference model without VFA and HOMA2-IR. Separate attenuation proportions were calculated for Model 3 (only VFA), Model 4 (only HOMA2-IR), and Model 5 (VFA combined with HOMA2-IR) for stage 4 CKM syndrome.
