# Supplementary material for: Non-lean versus lean MASLD: associations with advanced cardiovascular-kidney-metabolic syndrome in adults with type 2 diabetes mellitus, a cross-sectional study
Source: Front Endocrinol (Lausanne). 2026 Aug 7;17:1910398. doi: 10.3389/fendo.2026.1910398 (PMC13493292; doi:10.3389/fendo.2026.1910398)
Supplement: Supplementary file 3 [file Table3.docx]

**Table S3 Sensitivity Analysis: Sex-Stratified Odds Ratios for CKM Syndrome Comparing Lean and Non-lean Patients with MASLD and T2DM**

|  | ORs (95% CI) | | | | P value |
| --- | --- | --- | --- | --- | --- |
|  | Lean MASLD | Non-lean MASLD | | |  |
| CKM stage≥3 |  |  |  |  |  |
| male |  |  |  |  |  |
| Model 1 | 1 | 1.952(1.038-3.671) | | | 0.038 |
| Model 2 | 1 | 1.710(0.803-3.639) | | | 0.164 |
| Model 3 | 1 | 1.562(0.716-3.406) | | | 0.263 |
| female |  |  |  |  |  |
| Model 1 | 1 | 1.755(1.019-3.020) | | | 0.042 |
| Model 2 | 1 | 2.217(0.926-4.889) | | | 0.075 |
| Model 3 | 1 | 2.326(0.944-5.732) | | | 0.066 |
| CKM stage 4 |  |  |  |  |  |
| male |  |  |  |  |  |
| Model 1 | 1 | 2.073(1.089-3.945) | | | 0.026 |
| Model 2 | 1 | 2.195(1.041-4.628) | | | 0.039 |
| Model 3 | 1 | 2.971 (1.150-7.681) | | | 0.025 |
| female |  |  |  |  |  |
| Model 1 | 1 | 2.898(1.596-5.261) | | | ＜0.001 |
| Model 2 | 1 | 4.339(1.208-15.579) | | | 0.024 |
| Model 3 | 1 | 4.102(1.041-16.155) | | | 0.044 |

Model 1: Adjusted for age, sex, DD, smoking status, alcohol intake, dyslipidemia and hypertension.

Model 2: Further adjustment for use of LLDs, IIAs, OADs, SBP, DBP, and VFA.

Model 3: Further adjustment for TG, HDL-C, LDL-C, SUA, FIB-4 and HOMA2-IR.
